# "Scleroderma linearis: hemiatrophia faciei progressiva (Parry-Romberg syndrom) without any changes in CNS and linear scleroderma "en coup de sabre" with CNS tumor

**DOI:** 10.1186/1471-2377-9-39

**Published:** 2009-07-27

**Authors:** Beata Bergler-Czop, Anna Lis-Święty, Ligia Brzezińska-Wcisło

**Affiliations:** 1 Department of Dermatology Silesian Medical University in Katowice, Francuska Street 20/24, 40-027 Katowice, Poland

## Abstract

**Background:**

Hemifacial atrophy (Parry-Romberg syndrome) is a relatively rare disease. The etiology of the disease is not clear. Some authors postulate its relation with limited scleroderma linearis. Linear scleroderma "en coup de sabre" is characterized by clinical presence of most commonly one-sided linear syndrome. In a number of patients, neurological affection is the medium of the disease. The treatment of both scleroderma varieties is similar to the treatment of limited systemic sclerosis.

**Case presentation:**

We present two cases of a disease: a case of a 49-year-old woman with a typical image of hemifacial atrophy, without any changes of the nervous system and a case of a 33-year-old patient with an "en coup de sabre" scleroderma and with CNS tumor.

**Conclusion:**

We described typical cases of a rare diseases, hemifacial atrophy and "en coup de sabre" scleroderma. In the patient diagnosed with Parry-Romberg syndrome, with Borrelia burgdoferi infection and with minor neurological symptoms, despite a four-year case history, there was a lack of proper diagnosis and treatment.

In the second patient only skin changes without any neurological symptoms could be observed and only a precise neurological diagnosis revealed the presence of CNS tumor.

## Background

Hemifacial atrophy (Parry-Romberg syndrome) is a relatively rare disease. The etiology of the disease is not clear. Some authors postulate its relation with limited scleroderma linearis [1,2]. In a number of patients, neurological affection is the medium of the disease. Cory et al. [3] consider the origin of the disease to be a non-infectious, one-sided inflammatory process, connected with chronic, vasomotoric disturbances and with sympathetic nerves inflammation. Trauma appear in some patients. In some cases, the disease had of hereditary and autosomal character [1,3]. Clinically it comes to a face deformation, including skin, subcutaneous tissue and bones. A face asymmetry becomes visible. One side of a face seems smaller. The affected side eyeball is situated deeper, the mouth angle is lifted. The skin changes can include the hairy skin of the head, eyebrows and lashes, resulting in baldness focuses formation. The skin within the changes is of an increased compactness, tension and often it becomes over-colored or decolorized. Some patients suffer from headaches, which can be located in the area of trigeminal nerve innervations, and from temporary sensor disturbances [4]. Focal epilepsy on the opposite side can sometimes be diagnosed after neurological examination [1].

Histopathological examination proves fibrosis of the skin and adipose tissue atrophy without any features of an early inflammatory infiltration.

This disease is a type of condition which expires throughout many years. Deformations however, are permanent.

The connections of Parry-Romberg syndrome with scleroderma linearis or with en coup de sabre – typed limited scleroderma are often discussed. Some authors consider these to be two different diseases. For some however, hemifacial atrophy is an intensified form of linear scleroderma [1,5].

Linear scleroderma "en coup de sabre" is characterized by clinical presence of most commonly one-sided linear syndrome. The frontal region, from the eyebrows to the hairy skin of a head is the most typical location. The skin within the changes is of waxy-yellow color. On the head's hairy skin, linear focuses of cicatricial baldness appear. The progression of subsiding changes within the skin and hypodermis can lead to cranial bone deformation, to changes in the central nervous system tissue and to neurological symptoms alike in Perry-Romberg syndrome. The histological image of both diseases is similar. However, in case of "en coup de sabre" scleroderma, a massive lymphocytic inflammatory infiltration around the vessels of surface and deep plexuses of skin [1,6] can be observed in an early stage.

The treatment of both scleroderma varieties is similar to the treatment of limited systemic sclerosis. Penicillin and other antibiotics are applied. Braun-Falco et al. [1] provide a scheme based on antimalarial medicines: chloroquine and hydrochloroquine (1 pill/day) for 3–6 months. In severe cases drugs applied are: corticosteroids generally, retinoids, cyclosporine, cyclophosphamide, methotrexat. Locally – emollients, vitamin D analogs, light therapy – PUVA, UVA1 (10–50 J/cm2). When the pathological process is over, good effects are achieved by plastic surgery, including injection of autologic adipose tissue, synthetic collagen, filling preparation, containing hialuronic acid [1,5].

We present two cases of a disease: a case of a 49-year-old woman with a typical image of hemifacial atrophy, without any changes of the nervous system and a case of a 33-year-old patient with an "en coup de sabre" scleroderma and with CNS tumor.

We have approval of ethics committee of Silesian Medical University (ref. 1278/2008).

## Case presentation

### Case1

A female patient 49-year-old, cook. First discreet skin changes, with increased compactness and hyperpigmentation of the right side of the face, were noticed about 4 years ago by the patient. Astringing skin sensation and periodic, weakly intensified pains in the right side of the face and head, treated as a migraine, accompanied the appearance of the changes. The patient did not go through any injuries and did not complain about other neurological aliments. The skin symptoms were disregarded by the patient and were treated as a variant of a regular condition (?!). Because of that the patient was not diagnosed or treated. Nobody from the patient's surroundings and the general practitioners noticed the progressing face asymmetry. The patient is not suffering from any other chronic diseases and does not take any medicine on a regular basis. The family history towards autoimmunological and neurological diseases was negative. In July 2008 the patient was admitted to our Clinic with suspected hemifacial atrophy ("en coup de sabre" scleroderma?).

**Figure 1:**
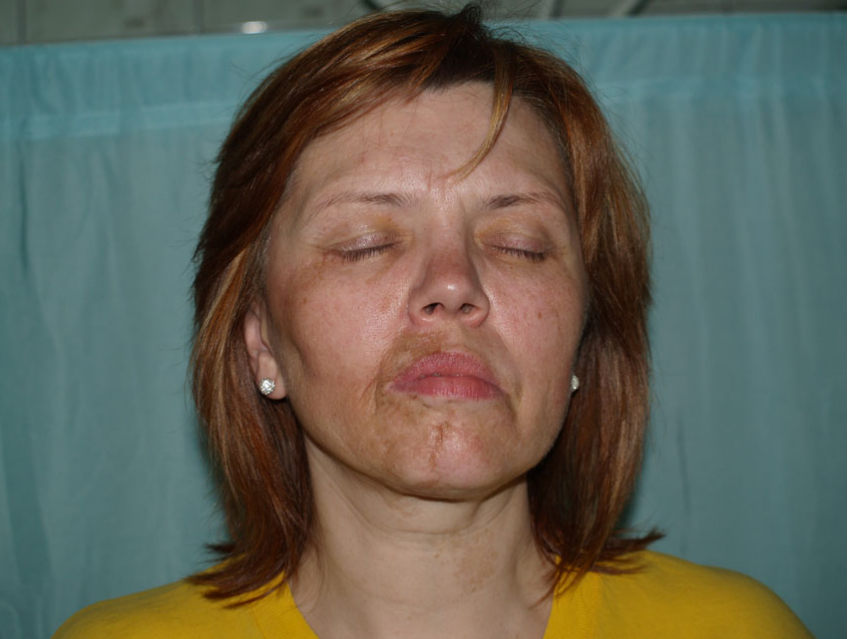
**A female patient 49-year-old, strongly distinct face asymmetry – the right side of the face was clearly smaller**.

**Figure 2:**
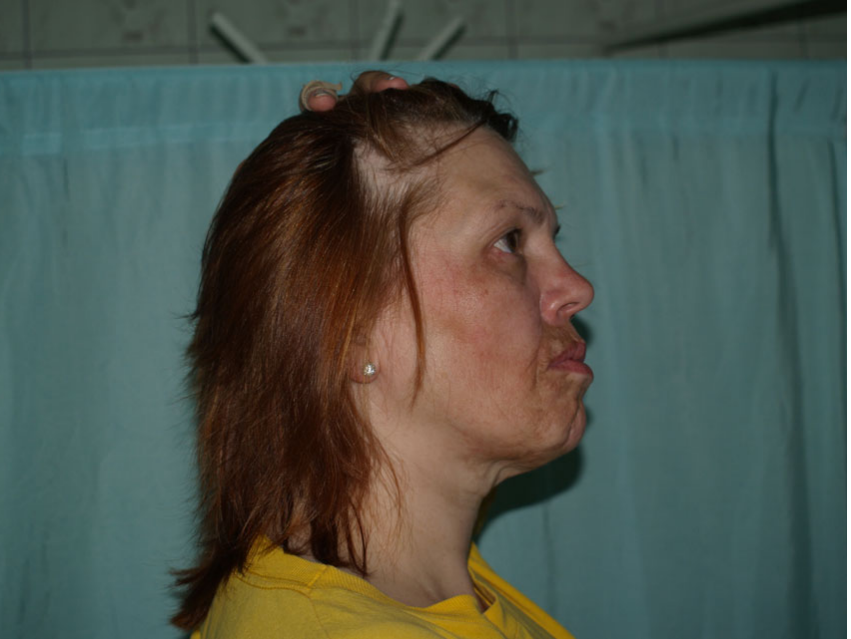
**A female patient 49-year-old, linearly arranged, oval, sunken baldness focuses on the head's hairy skin, near the right temple**.

Clinically, in admission to the hospital, strongly distinct face asymmetry could be observed. The right side of the face was clearly smaller. The mandible angle was lifted and smoothed. The lip redness on the affected side became thin and lifted. The cheek was sunken. The eyeball was situated deeper. The skin surrounding the changes was more coherent, thin and strongly seal-colored. There were linearly arranged, oval, sunken baldness focuses on the head's hairy skin, near the right temple. Skin surrounding these focuses was unchanged. The mucous and genitals membranes and nail plates were normal. The peripheral lymph glands – not enlarged (fig. 1,2).

In the performed laboratory tests (morphology with smear, fasting glucoses level, glycemia profile, AspAT, AlAT, bilirubin, GGTP, alkaline phosphatase, creatinine, urea, uric acid, protein with electrophoresis, ASO, Latex-R, Waaler-Rose test, VDRL, general urinalysis with culture) no aberrations were found.

Antigens against Borrelia burgdorferi in IgM class with a 1,26 titre (positive, norm up to 1,1) were found in blood serum. IgG antibodied – negative. Penicillin test – positive.

Histopathological examination of the right cheek change revealed traits of fibrosis, collagen fibers thickening, skin edema and skin adnexa and vessels atrophy, without an inflammatory infiltration. Image examination – x-ray of the chest, upper part of the digestive tract, abdominal cavity USG were all normal. Capillaroscopy – within the norm.

Head CT performed in a spiral technique, in transverse section, in forehead and fibular reconstructions, before and after the intravenous administration of contrast medium revealed only a bone septum on the right side of the sphenoidal sinus and the mucous membrane thickening within its area. The encephalon image – normal. NMR of the head – without pathology. Electromyography (the muscles end the nerves of the face: the right and left roundabout muscle of the mouth, the right and left facial nerve) – in the right roundabout muscle of the mouth the small impoverishment of the effort record. Conducition in both facial nerves without pathology. With an indirect, immunofluorescent method on Hep2 cells, no ANA antigen antibodies were found. Mycological examination on skin changes was also negative. Laryngological, dental consultation have not shown any abnormalities.

Neurological examination confirmed one-sided headaches in patient's anamnesis, without any focal symptoms of CNS damages. Suggested performing head CT and NMR and EMG (results a.b.).

During the patient's stay at the Clinic, cefuroxym (Plixym) in a dose of 2 × 1,5 g iv for 10 days was applied, continuing the treatment with oral cefuroxym (Zinnat) 2 × 250 mg for 4 weeks. Emollients and low-energy laser was applied locally (15 procedures). The patient was sent to the Infectious Diseases Outpatient Clinic in order to have a Borrelia burgdorferi infection confirmed with the use of Western-blot method (positive). Currently, the patient is under supervision of Dermatological Outpatient Clinic in order to continue the treatment and a possible committal to plastic surgery treatment after the pathological process is over.

### Case 2

**Figure 3:**
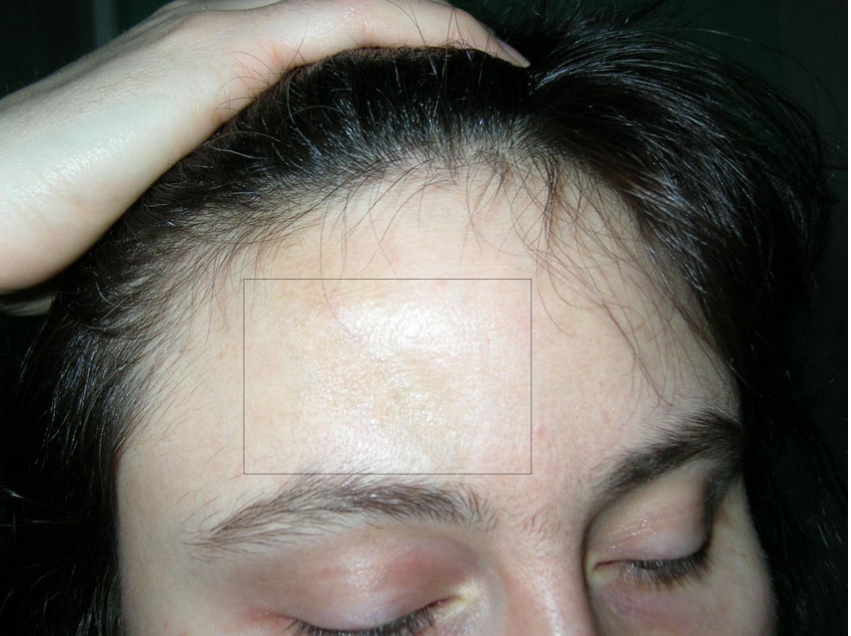
**33-year-old patient, woman – on the right side of the forehead, linear, ivory-colored scar with skin and hypodermis atrophy and bone recess**.

33-year-old patient, a teacher. First, slightly visible skin changes appeared, with a linear structure, increased compactness and with skin atrophy within the right side of the forehead, they were noticed by the patient about 2 years before. The appearance of these changes was not accompanied by any subjective symptoms. The patient did not go through any injuries and did not complain about other neurological aliments. Because of this, the patient was not diagnosed or treated. The patient is not suffering from any other chronic diseases and does not take any medicine on a regular basis. The family history towards autoimmunological and neurological diseases was negative. In February 2007 the patient was admitted to our Clinic with suspected circumscribed scleroderma, "en coup de sabre" – type. When admitting to the hospital, observed skin changes included the forehead and head's hairy skin. On the right side of the forehead, linear, ivory-colored scar change could be seen, with skin and hypodermis atrophy and bone recess. The scar was reaching the head's hairy skin. In the right parieto-temporal area, linearly arranged, oval, recessed focuses of baldness were located. The skin around these changes was unchanged. The mucous of oral cavity, genital area and nail plates were normal. The peripheral lymph glands – not enlarged (fig. 3).

**Figure 4:**
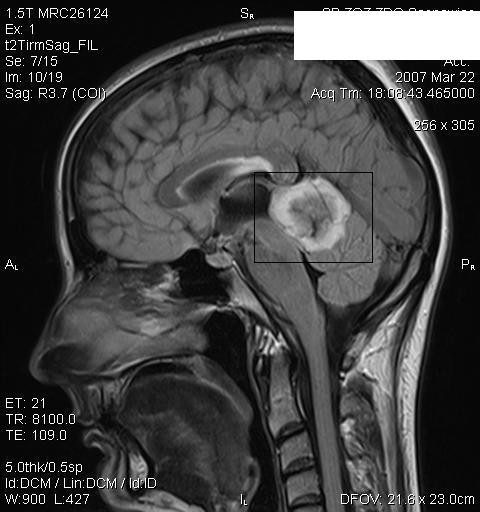
**33-year-old patient, woman, MRI and MRI spectroscopy suggest the presence of a change in a form of teratoma**.

In laboratory examination no aberrations found (morphology with smear, fasting glucoses level, glycemia profile, AspAT, AlAT, bilirubin, GGTP, alkaline phosphatase, creatinine, urea, uric acid, protein with electrophoresis, ASO, Latex-R, Waaler-Rose test, VDRL, general urinalysis with culture). No antigens against Borrelia burgdorferi in IgM and IgG class found in blood serum. Penicillin test – positive. Imagine examination – x-ray of the chest, upper part of the digestive tract, abdominal cavity USG were all normal. Capillaroscopy – within the norm. Head CT-performed in a spiral technique, in transverse aspect, before and after the intravenous administration of contrast medium. The CT revealed: a extracerebral tumor located subtentorially (in the upper part), towards the back from the callous body and from the III cerebral ventricle, without any clear features of growing into these structures. The change seems to be adjacent to the tegmentum of midbrain to the inferior direction and adjoins the cerebellum in the inferior and posterior directions. Tumor, which is of about 28 × 26 × 24 mm, is heterogeneous, with a large amount of calcifications and does not show any features of contrast-induced augmentation. It is slightly modeling the straight sinus in the inferior direction, with no evident features of its infiltration. In the anterior direction the tumor seems not to be reaching the pineal gland area, in the pineal gland area visible minor calcifications. The supratentorial ventricular apparatus is not enlarged, the CT does not clearly show cerebral aqueduct, the IV cerebral ventricle is within the norm. CT does not show any other changes. Conclusions: tumor like, extracerebral structure demanding further diagnosis in MRI examination. NMR of the head: expansive process located posterioly to the pineal gland near the upper vermis of cerebellum. Brain angiography – no pathological vascularization features. MRI and MRI spectroscopy suggest the presence of a change in a form of teratoma (fig. 4).

Beta-HCG – within the norm. AFP – within the norm. With an indirect, immunofluorescent method on Hep2 cells, no ANA antigen antibodies were found. Mycological examination on skin changes was also negative. Neurological consultation did not reveal any focal signs of OUN injury. Neurosurgical consultation was suggested, due to the presence of an extracerebral tumor in the CT examination of the head (Osteoma? Cylindoma?)

During the patient's stay at the Clinic, an oral antibiotic therapy with cefuroxym (Zinnat) 2 × 250 mg for 14 days was applied. Emollients were applied locally. The patient was sent to the Neurosurgical Clinic for further diagnosis and for the extracerebral tumor treatment.

On account of suspected *teratoma*, the lack of intracranial hypertension and signs of focal CNS injury, the Neurological Clinic decided to temporarily put the medical procedure on hold.

Currently, the patient is under the supervision of the Dermatological Clinic in order to continue the treatment and a possible committal to plastic surgery treatment after the pathological process is over.

## Discussion

Hemifacial atrophy (Parry-Romberg syndrome) is a relatively rare disease. "En coup de sabre" scleroderma is more common, which, by some authors, is treated as variously intensified forms of the same nosologic unit. The etiology of both diseases is not clear [1].

Frequent coexistence of skin changes within hypodermis and bones of face, with neurological symptoms and with structural and functional changes of CNS are uncontested. Braun – Falco et al. [1] claim, that in most patients pains within trigeminal nerve innervations occur before visible deformations appear. In case of our first patient, the anamnesis, for many years showed there were weakly intensified, one-sided headaches, treated as migraine. Neurological treatment however, did not show any deviation.

The second patient did not report any subjective symptoms which would accompany skin changes. Neurological treatment also did not show any deviation. CT however, revealed the presence of CNS tumor located subtentorially (in the upper part), towards the posterior side from the callous body and from III cerebral ventricle, without any clear features of growing into these structures.

Aynaci et al. [7] described a child with hemifacial atrophy features without any neurological deficiency, with Adie's syndrome on the affected side of the face – with mydriasis, no reaction to light, with a slow reaction to convergence and accommodation (tonic pupil). Schnitzler et al. [8] introduced a 25-year-old patient. At the age of 14, he had a tonic pupil on the right side and epilepsy attacks. At the age of 22, the patient noticed progressive atrophy of the right side of his face. Authors postulate the common, immunological etiology of hemifacial atrophy, Adie's syndrome and epilepsy.

The literature informs that in many patients changes in CNS, visible in CT and NMR appear. Both examinations performed on our patient with diagnosed Perry-Romberg syndrome were normal. The changes described above were found in the patient with „en coup de sabre" scleroderma.

Unterberger et al. [6] introduced a case of a 24-year-old patient, with „en coup de sabre" scleroderma and with focuses of scleroderma linearis. In the 33^rd ^week of the patient's pregnancy, right hemiplegia symptoms appeared, with a positive Babinski's sign on the affected side. The symptoms escalated significantly after the labor (caesarean section). NMR of the head revealed numerous focuses of an increased signal in the left hemisphere, spreading towards the fronto-temporal area, including the white substance, with minor subcortical hyper intensive focuses from the right frontal side.

In case of the child described by Cory et al. [3], the symptoms of hemifacial atrophy were developing for a 20 month period. The tomography and the magnetic resonance revealed numerous, one-sided focuses of infarcts in the amygdaloid body, multiple deep and subcortical changes of the signal in the white substance and a mild thinning of the cortex. The angiographic examination was normal. Okumura et al. [9] performed precise pictorial examination of the patient with Parry-Romberg syndrome. Focuses of an increased condensation in the white substance of the left hemisphere were described in NMR. In the proton spectroscopy, a form of NMR, no aberrations has been found. Monoprotonic, emissive tomography showed an increase of blood perfusion in the left hemisphere. These results correlate with the aberrations in the clinical research and with hemifacial atrophy signs. In the case of a 32-year-old patient described by Moon et al. [4], with diagnosed Perry-Romberg syndrome, except for the visible face asymmetry, intermittent headaches occurred, accompanied by sensory disturbances affecting the hearing and the sight. NMR revealed a mild asymmetric atrophy of the right hemisphere and few nonspecific white substance condensations on the affected side. DTI (Diffusion tensor imaging) and the tractography confirmed the changes in the nervous fibers of the white substance sensory tracts on the right hemisphere. Sathornsumetee et al. [10] introduced a case of a 4-year-old boy with a progressive hemifacial atrophy, which was accompanied by multiple neurological symptoms. The patient was diagnosed with epilepsy, progressive hemisphere atrophy and severe changes within the brain stem. Paprocka et al. [5] introduced a 10-year-old girl patient, with Rasmussen's encephalitis, which is connected with a chronic inflammatory condition and with a damage of one, intact hemisphere. Epilepsy focuses and one-sided neurological symptoms are observed clinically. Right-sided hemiparesis was observed in the patient. From the age of 2, the patient was diagnosed with left hemifacial atrophy and with a focus of „en coup de sabre” linear scleroderma within the forehead. The authors examine the potential relation of linear scleroderma, hemifacial atrophy and described Rasmussen's encephalitis. In 2003 Stone [11] performed an internet research on 205 patients with Parry-Romberg syndrome and found 11% with epilepsy and 19% with limb motor activity changes. Błaszczyk et al. [2] examined 19 patients with signs of progressive hemifacial atrophy and en coup de sabre scleroderma towards changes in CNS. Routine examination, EEG, NMR, angio-NMR and 99 mTc-HM-PAO-SPECT were performed. The examinations revealed significantly increased frequency of change occurrence in CNS in patients with hemifacial atrophy and en coup de sabre scleroderma signs than in the control group. Gambichler et al. [12] introduced a 23-year-old patient with two-sided en coup de sabre scleroderma and with a left-sided hemifacial atrophy. The patient also suffers from epilepsy attacks and a paralysis of oculomotor nerves and of right-sided facial nerve. The authors suggest a close relation between en coup de sabre scleroderma, hemifacial atrophy and neurological symptoms in the patient.

In our first patient examined, antibodies against Borrelia burgdofei in IgM class were found. The infection was confirmed with Western-blot method. It is difficult however, to talk about the relation of a fresh infection with lasting for over 4 years progressive process of facial atrophy. This is rather a coexistence of both diseases. Sommer et al. [13] research also did not confirm the relation of Borrelia burgdofei infection and the progressive facial atrophy. 278 patients took part in this research.

Relatively few reports concern the treatment of hemifacial atrophy and "en coup de sabre" scleroderma linearis. Most authors use similar medicines as in the treatment of scleroderma: procaine penicillin and other antibiotics, anti-malarial medicine, corticosteroids generally, retinoids, cyclosporine, cyclophosphamide, methotrexat. Locally – emollients, vitamin D analogs, light therapy – PUVA, UVA1 (10–50 J/cm2). This treatment however, aims to speed up the pathological process towards its end, because, in fact there is no medicine for an effective therapy of both scleroderma and Perry-Romberg syndrome. Tollefson et al. [14] used anti-malarial medicine in 57,1% of patients and methotrexat in 28,6% of patients with hemifacial atrophy and "en coup de sabre" linear scleroderma. The rest of the patients were only taking emollients. The effectiveness in all groups was controversial.

The described patient, without changes in CNS received cefuroxym in a dose of 2 × 1,5 g iv for 10 days, continuing the treatment with oral cefuroxym (Zinnat) for 4 weeks. In the case of the second patient, cefuroxym in a dose of 2 × 1,5 g iv for 10 days was applied and the patient was sent to neurosurgical treatment.

After the pathological process is over, good effects are achieved by plastic surgery, including injection of autologic adipose tissue, synthetic collagen, filling preparation, containing hialuronic acid [1,5,15].

These techniques are planned to be implemented in the future.

## Conclusion

We described typical cases of a rare diseases, hemifacial atrophy and "en coup de sabre" scleroderma. In the patient diagnosed with Parry-Romberg syndrome, with Borrelia burgdoferi infection and with minor neurological symptoms, despite a four-year case history, there was a lack of proper diagnosis and treatment.

In the second patient only skin changes without any neurological symptoms could be observed and only a precise neurological diagnosis revealed the presence of CNS tumor.

## Consent

We confirm that written consent was obtained from the patient or their relatives for publication of study and the use of any images.

## Competing interests

The authors declare that they have no competing interests.

## Authors' contributions

This manuscript was draft by BBC, ALŚ, LBW. BBC – main conception, design, acquisition of data, interpretation of data, writing; ALŚ – writing assistance, acquisition of data; LBW – references, writing assistance. All authors contributing to its critical review and approving the final draft.

## Pre-publication history

The pre-publication history for this paper can be accessed here:



## References

[B1] Braun-Falco O, Plewig G, Wolff HH, Braun-Falco O, Plewig G, Wolff HH, et al (2002). Connective tissue diseases. Dermatology, Czelej, Lublin.

[B2] Błaszczyk M, Królicki L, Krasu M (2003). Progressive facial hemiatrophy: central nervous system involvement and relationship with scleroderma en coup de saber. J Rheumatol.

[B3] Cory RC, Clayman DA, Faillace WJ (1997). Clinical and radiologic findings in progressive facial hemiatrophy (Parry-Romberg syndrome). Am J Neuroradiol.

[B4] Moon WJ, Kim HJ, Roh HG (2008). Diffusion tensor imaging and fiber tractography in Parry – Romberg syndrome. Am J Neuroradiol.

[B5] Paprocka J, Jamroz E, Adamek D (2006). Difficulties in differentiation of Parry-Romberg syndrome, unilateral facial sclerodermia, and Rasmussen syndrome. Childs Nerv Syst.

[B6] Unterberger I, Trinka E, Engelhardt K (2003). Linear scleroderma "en coup de sabre" coexisting with plaque-morphea: neuroradiological manifestation and response to corticosteroids. J Neurol Neurosurg Psychiatry.

[B7] Aynaci FM, Sen Y, Erdol H (2001). Parry – Romberg syndrome associated with Adie's pupil and radiologic findings. Pediatr Neurol.

[B8] Schnitzler ES, Michelson G, Harazny J (2003). Hemiatrophia faciem progressiva and tonic pupil. Klin Monatstbl Augenheilkd.

[B9] Okumura A, Ikuta T, Tsuji T (2006). Parry – Romberg syndrome with a clinically silence white master lesion. Am J Neuroradiol.

[B10] Sathornsumetee S, Schanberg L, Rabinovich E (2005). Parry-Romberg syndrome with fatal brain stem involvement. J Pediatr.

[B11] Stone J (2003). Parry-Romberg syndrome: a global survey of 205 patients using the Internet. Neurology.

[B12] Gambichler T, Kreuter A, Hoffman K (2001). Bilateral linear scleroderma "en coup de sabre" associated with facial atrophy and neurological complications. BMC Dermatol.

[B13] Sommer A, Gambichler T, Bachrach-Buhles M (2006). Clinical and serological characteristics of progressive facial hemiatrophy: a case of 12 pateints. J Am Acad Dermatol.

[B14] Tollefson MM, Witman PM (2007). En coup de saber morphea and Parry-Romberg syndrome: a retrospective review of 54 patients. J Am Acad Dermatol.

[B15] Roller E, Reifenberger J, Homey B (2006). Hemiatrophia faciei progressiva (Parry-Romberg-syndrome). Hautarzt.

